# Crowdsourcing Malaria Parasite Quantification: An Online Game for Analyzing Images of Infected Thick Blood Smears

**DOI:** 10.2196/jmir.2338

**Published:** 2012-11-29

**Authors:** Miguel Angel Luengo-Oroz, Asier Arranz, John Frean

**Affiliations:** ^1^Biomedical Image Technologies groupDIE, ETSI TelecomunicaciónUniversidad Politécnica de Madrid, CEI Moncloa UPM-UCMMadridSpain; ^2^Biomedical Research Networking Center in Bioengineering, Biomaterials and NanomedicineCIBER-BBNMadridSpain; ^3^Nebutek Soluciones SLVizcayaSpain; ^4^National Institute for Communicable Diseases, National Health Laboratory ServiceJohannesburgSouth Africa; ^5^School of Pathology, Faculty of Health Sciences, University of the WitwatersrandJohannesburgSouth Africa

**Keywords:** Crowdsourcing, Malaria, Image Analysis, Games for Health, Telepathology

## Abstract

**Background:**

There are 600,000 new malaria cases daily worldwide. The gold standard for estimating the parasite burden and the corresponding severity of the disease consists in manually counting the number of parasites in blood smears through a microscope, a process that can take more than 20 minutes of an expert microscopist’s time.

**Objective:**

This research tests the feasibility of a crowdsourced approach to malaria image analysis. In particular, we investigated whether anonymous volunteers with no prior experience would be able to count malaria parasites in digitized images of thick blood smears by playing a Web-based game.

**Methods:**

The experimental system consisted of a Web-based game where online volunteers were tasked with detecting parasites in digitized blood sample images coupled with a decision algorithm that combined the analyses from several players to produce an improved collective detection outcome. Data were collected through the MalariaSpot website. Random images of thick blood films containing *Plasmodium falciparum* at medium to low parasitemias, acquired by conventional optical microscopy, were presented to players. In the game, players had to find and tag as many parasites as possible in 1 minute. In the event that players found all the parasites present in the image, they were presented with a new image. In order to combine the choices of different players into a single crowd decision, we implemented an image processing pipeline and a quorum algorithm that judged a parasite tagged when a group of players agreed on its position.

**Results:**

Over 1 month, anonymous players from 95 countries played more than 12,000 games and generated a database of more than 270,000 clicks on the test images. Results revealed that combining 22 games from nonexpert players achieved a parasite counting accuracy higher than 99%. This performance could be obtained also by combining 13 games from players trained for 1 minute. Exhaustive computations measured the parasite counting accuracy for all players as a function of the number of games considered and the experience of the players. In addition, we propose a mathematical equation that accurately models the collective parasite counting performance.

**Conclusions:**

This research validates the online gaming approach for crowdsourced counting of malaria parasites in images of thick blood films. The findings support the conclusion that nonexperts are able to rapidly learn how to identify the typical features of malaria parasites in digitized thick blood samples and that combining the analyses of several users provides similar parasite counting accuracy rates as those of expert microscopists. This experiment illustrates the potential of the crowdsourced gaming approach for performing routine malaria parasite quantification, and more generally for solving biomedical image analysis problems, with future potential for telediagnosis related to global health challenges.

## Introduction

Crowdsourcing methodologies leveraging the contributions of citizen scientists connected via the Internet have recently proved to be of great value to solve certain scientific challenges involving “big data” analysis that cannot be entirely automated [1]. In the GalaxyZoo project, citizen scientists classified imagery of hundreds of thousands of galaxies drawn from the Sloan Digital Sky Survey and the Hubble Space Telescope archive [2]. Crowdsourced contributions can be achieved with different motivation strategies, such as micropayments or games. The “serious games” concept refers to an intention not only to entertain users, but also to train or educate them [3]. The “gamification” [4] of the crowdsourcing approach enables a higher motivation of the participants and, using the Internet as a vehicle, untaps an underexploited resource for scientific research [5,6]: it is estimated that 3 billion hours per week are spent playing computer and videogames worldwide [7]. For instance, Fold-It, an online game where players solve 3-dimensional puzzles by folding protein structures, has resulted in several breakthrough scientific discoveries [8-10]. Another recent growing trend is the use of crowdsourcing techniques for participatory health research studies in which individuals report in real time a variety of health conditions [11], providing a promising complement to traditional clinical trials. Considering crowdsourced image analysis, collective processing has been recently explored for earthquake damage assessment from remote sensing imagery [12]. However, this methodology has not yet been mainstreamed for biomedical image analysis.

In this context, analysis of microscopic images of malaria-infected blood samples is an appealing goal. Worldwide, there are more than 200 million malaria cases and approximately 800,000 deaths annually, mainly in children [13,14]. Careful optical microscopic examination of a well-stained blood film remains the gold standard for malaria diagnosis [15]. Confirmation of a negative diagnosis is ultimately dependent on the technician’s expertise and can take up to 20 minutes. In addition, as malaria prevalence decreases in one specific place over time, microscopy technician skills may now be needed in other regions. Fast, cheap, ubiquitous, and accurate diagnosis is a priority in the Agenda for Malaria Eradication [16]. Although automated processing methodologies have been used extensively for the analysis of digitized blood smears [17,18], currently there are no completely automated image processing systems that can achieve perfect parasite recognition [19-24]. The main problem in computer-aided malaria diagnosis is that algorithms are usually not very robust with respect to the variable appearance of the parasites and changing image acquisition conditions.

The goal of this research was to test the feasibility of a crowdcomputing approach for malaria parasite quantification in which nonexperts count parasites in digitized thick blood smears through an online game (crowdsourcing) and a decision algorithm combines the data generated by several players in order to achieve a collective detection with a higher accuracy rate than an individual analysis. This idea—gaming for distributed malaria image analysis—has been also explored in a recent study by Mavandadi et al [25], in parallel to and independently of this study. These researchers designed a video game and a processing pipeline to investigate whether nonexperts can assess if a single-cell image extracted from a digitized thin blood sample is infected with malaria or not. Although this study and the present research share a similar vision and goal, the research questions posed and solutions adopted differ substantially in terms of the data analyzed, the nature of the participants, the main task required of them, and the processing methodologies.

The proposed system in this study provides a new tool for parasite counting, but not malaria diagnosis, which is a more complex problem [26]. For this purpose, the microscopist protocol will need to be translated completely into a gaming protocol, including assessing the presence or absence of parasites, the parasite species, and growth stages and prognostic markers, such as schizonts or gametocytes, or pigment load. In the long run, crowdsourced remote telediagnosis from images acquired with optical microscopy and distributed worldwide through the Internet and possibly with systems that integrate the microscope into mobile phones [27,28], might have a potential impact for malaria-endemic countries because diagnosis availability and its cost could be optimized. However, in addition to the need for conventional laboratory processing and imaging equipment to prepare the material to a sufficiently high standard, this kind of analysis will require a communications infrastructure with enough bandwidth to distribute the images over the Internet and a critical number of online participants in order to ensure timely analysis of the images.

This work presents a proof-of-concept system that explores the feasibility of an online game-based, crowdsourced solution for malaria parasite quantitation in digitized images of thick blood smears.

## Methods

We selected an image database of malaria-positive blood films that had been previously analyzed by experts to generate gold standards. These images were then incorporated into an online game. The player’s task was to click on the parasites. When a player found all the parasites present in 1 image (constituting a level) within a limited amount of time, the game continued by presenting a new image. Otherwise, the game was over. All the players’ clicks were registered in a database. After 1 month, all the collected data was preprocessed in order to group all the clicks that players placed around the different objects in the image: parasites, white blood cells (leukocytes), and background noise. Finally, an algorithm that combined the different games to increase accuracy was developed and evaluated.

### Ethics Statement

The malaria images used in this research were previously used to evaluate automated image analysis methods [20]. Original blood samples and resultant test images were collected and used with ethical approval from the Human Research Ethics Committee (Medical), University of the Witwatersrand, Johannesburg, South Africa (protocol number M051126). No new ethical review board approval was required since the digital images used in our work were not linked to any patient data or diagnosis and were digitally shared for microscopic training evaluation purposes. The data analyzed in this research were anonymously produced by online volunteers who agreed to play an Internet game. The participants were informed of the research purposes of the game on the game webpage.

### Image Database

The image database was compiled from 28 Giemsa-stained thick films made from blood infected with malaria (*Plasmodium falciparum*) parasites, acquired using a 50× objective in a conventional laboratory optical microscope. Medium to low parasitemia images were selected for the game because of its design (1-minute games) and the fact that discrepancies between automatic counting methodologies and manual expert counting tend to be greater in low parasitemia cases. A gold standard mask image was generated for each of the 28 images to evaluate player performance.

### Game Architecture

**Figure 1:**
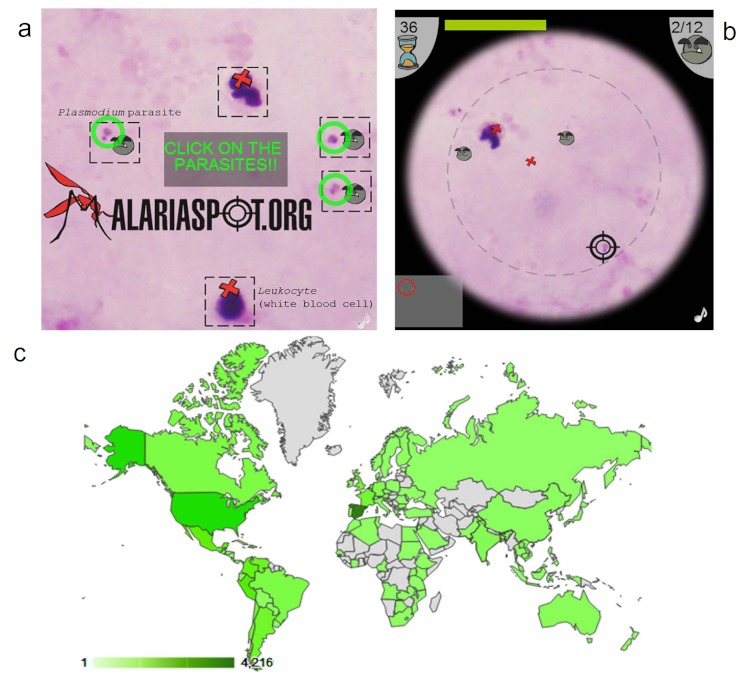
MalariaSpot example screens and player distribution. (a) Splash screen of MalariaSpot game website showing the game instructions. (b) Example of MalariaSpot game screen. (c) Map showing the geographic distribution of players during the evaluation period (the darkness of the country is proportional to the number of visits).

The objective of the MalariaSpot game was to tag as many parasites as possible in an image in 1 minute. The instructions—what is a parasite and what it is not—were briefly explained in the splash screen of the game website (Figure 1a). During the game, if the player found all the parasites in 1 image in the allowed time, a new image was presented (Figure 1b). Therefore, a player could analyze several images (levels) in a single game. In order to reinforce the game’s addictive nature, the players were given continuous feedback: each click was compared with the gold standard and an icon was placed immediately at the tag position to indicate a correct or incorrect selection. In addition, if the player misidentified an object and clicked in a wrong location (eg, on a leukocyte), the player was penalized by reducing the remaining time available to solve the level. Players were confronted with different, randomly selected test images. The difficulty of the levels increased as the time penalty for wrong tags grew with each level. As a motivation strategy, at the end of the game players were invited to register and provide their name, email address, and country in order to be included in the table of high scorers depicting the top daily, weekly, and monthly players.

### Data Collection

The MalariaSpot game webpage was launched on April 25, 2012 (World Malaria Day). During the following month, more than 6000 players from 95 different countries (Figure 1c) visited the game webpage according to the number of Internet Protocol (IP) addresses reported by Google Analytics, although the actual number of players was probably larger because those connecting from big institutions, such as universities, share the same IP address and other players may have blocked the Google Analytics script. Online volunteers played a total of 12,105 games that resulted in the analyses of 20,049 images and generated a database of 270,207 tags. Social media was the main traffic source; approximately 30% of the players originated from a Facebook link and 30% came from a Twitter reference. Most of the remaining visits were through links in digital newspapers and blogs, especially from Spanish-speaking countries.

### Data Preprocessing

**Figure 2:**
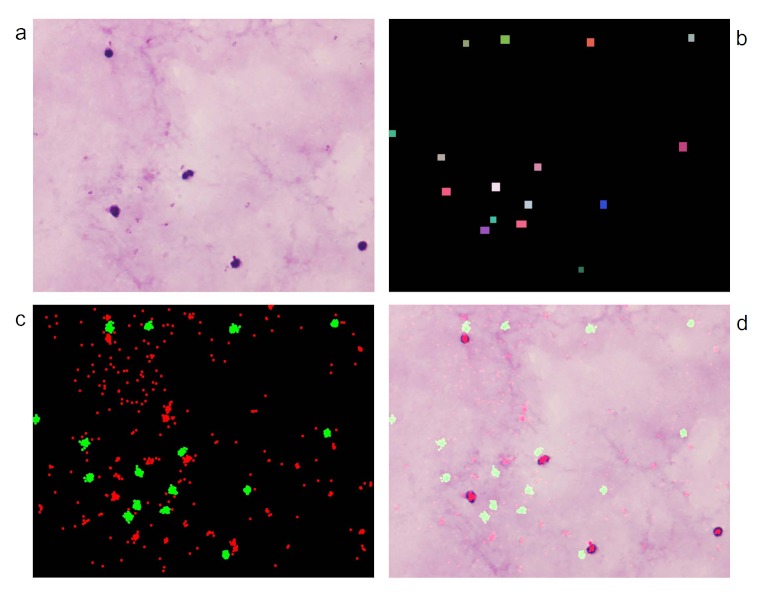
Crowdsourced image analysis of thick blood film infected with malaria. (a) Test image analyzed in the game. (b) Gold standard image in which each label corresponds to a parasite. (c) Aggregation of gamer’s clicks where green regions correspond to correctly tagged parasites and red regions to players’ mistakes. (d) Gamers’ clicks superimposed on raw image.

All the players’ clicks were saved into a database containing the user identification number, image identification number, x-position and y-position on the game screen, time of the click (from the start of the level), and whether the click was on a true parasite or not (see Figures 2a-d and Multimedia Appendix 1). In a preprocessing step, we generated a binary matrix, I_n_(g,p), for each test image *n*, where each row *g* contains a different game and each column *p* corresponds to a parasite. A value of 1 at a certain position, I_n_(g_i_,p_j_) = 1, means that the parasite with index *j* has been clicked in the game *i*. Otherwise, I_n_(g_i_,p_j_) = 0. The number of rows is the number of games that have been played at each test image *n*. The number of columns corresponds to the number of parasites for a given level in the gold standard plus the number of phantom parasites. We defined a phantom parasite as an object in the image that is not a parasite and that has been tagged by ≥ 1 players. The phantom parasites were defined in order to group together all the clicks that were around the same position, but not on the identical pixel (eg, all the clicks that were inside a leukocyte were considered to be pointing at the same phantom parasite). An image processing pipeline that grouped together clicks that were at a distance of less than the typical parasite size was implemented in order to generate all the connected components in each image that corresponded to phantom parasites (Figure 2c). Therefore, the output of this preprocessing stage consisted of 1 binary matrix per test image that characterized the performance of all the games played for each image. Additionally, filtered versions of these matrices were created by selecting only the data from games in which at least 1 level was completed, 2 levels were completed, and so on.

### Collective Parasite Detection: Quorum Algorithm

**Figure 3:**
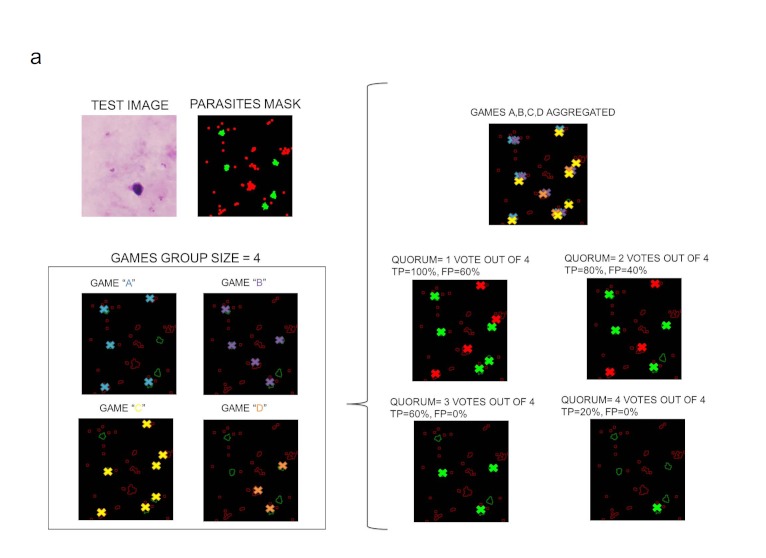
Illustrative example of the quorum algorithm. Parasite detection results on test image obtained from the combination of 4 games processed with different quorum values.

A critical aim of this research was to show how individual nonexpert analysis can be combined to achieve higher accuracy rates. In order to combine the games of several players and produce a single “detection,” we implemented a quorum algorithm. The output of the quorum collective detection features all the image objects (both true parasites and phantom parasites) in one image that have been tagged in at least *X* individual games out of a larger group of *Y* games (Figure 3). The idea is simple: an object is considered in the collective detection if it has been tagged (“voted”) in at least X out of Y (X ≤ Y) games. Typically, when the quorum value increases, there are fewer true positives and false negatives. In order to evaluate the performance of different group sizes and quorum values, we randomly selected 1000 subsets of games per (X,Y) couple with a maximum group size of Y = 30 games. For each individual subset of Y random games in 1 image, the collective detection performance was measured for all quorum values of X ≤ Y. Performance evaluation was also measured taking the subset of games that passed at least level 1, level 2, level 3, and level 4.

## Results

**Figure 4:**
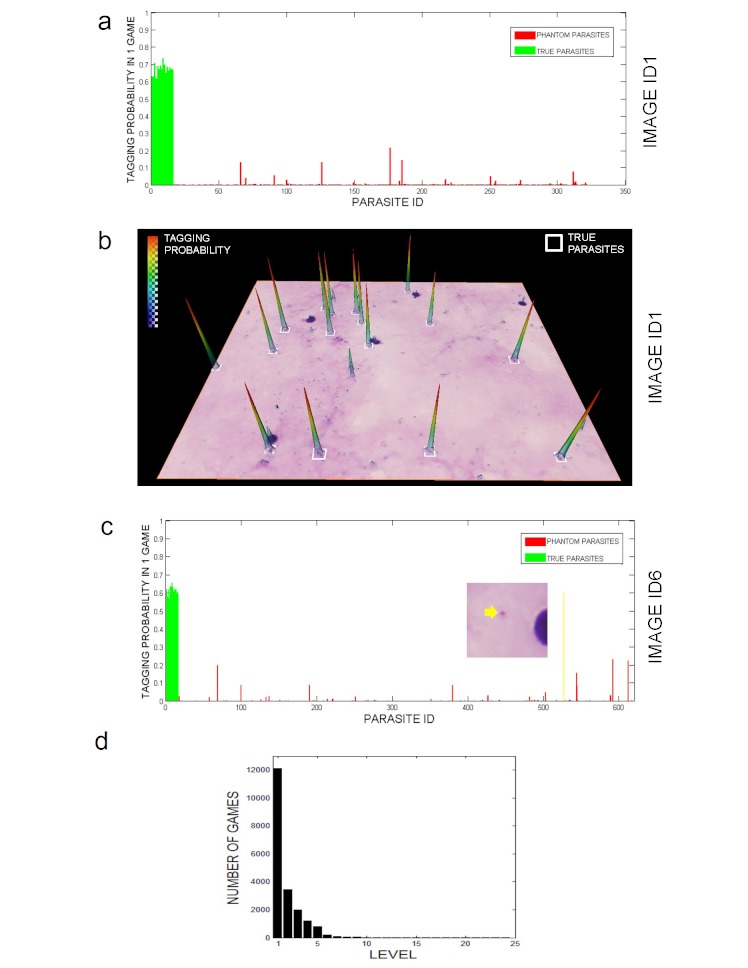
Individual gamer’s performance. (a) Tagging probability for true parasites and phantom parasites on image ID1 based on all games. (b) Tagging probability field superimposed on raw image. True parasites (gold standard) are signaled by white squares. (c) Aggregated tagging probability for true parasites and phantom parasites on image ID6. Note that the probability of the phantom parasite (tagged in yellow) is as high as the true parasites. Detailed analysis showed that it was a mistake on the gold standard. (d) Number of games played at each level.

Out of a total of 270,207 clicks, 78.65% tagged a true parasite. Analysis of the levels reached by the players reveals that approximately one-third of the players were able to find all the parasites in an image, independent of the level (see Figure 4d). Additionally, once players successfully completed level 5, they became game experts and no longer followed the 1 in 3 chance of passing to the next level—they could complete as many as 22 levels (achieved by the best player so far). Interestingly, the overall number of clicks on each of the parasites in 1 image was similar, meaning that although 2 of 3 players usually did not complete the level, all the parasites were equally difficult to identify (Figures 4a and b). This fact was corroborated in a special case for image ID6, where the probability of tagging one particular phantom parasite was as high as the typical probability for a true parasite. A further look into the gold standard revealed that, in fact, this phantom parasite was a true parasite that was not included in the gold standard by mistake (Figure 4c).

**Figure 5:**
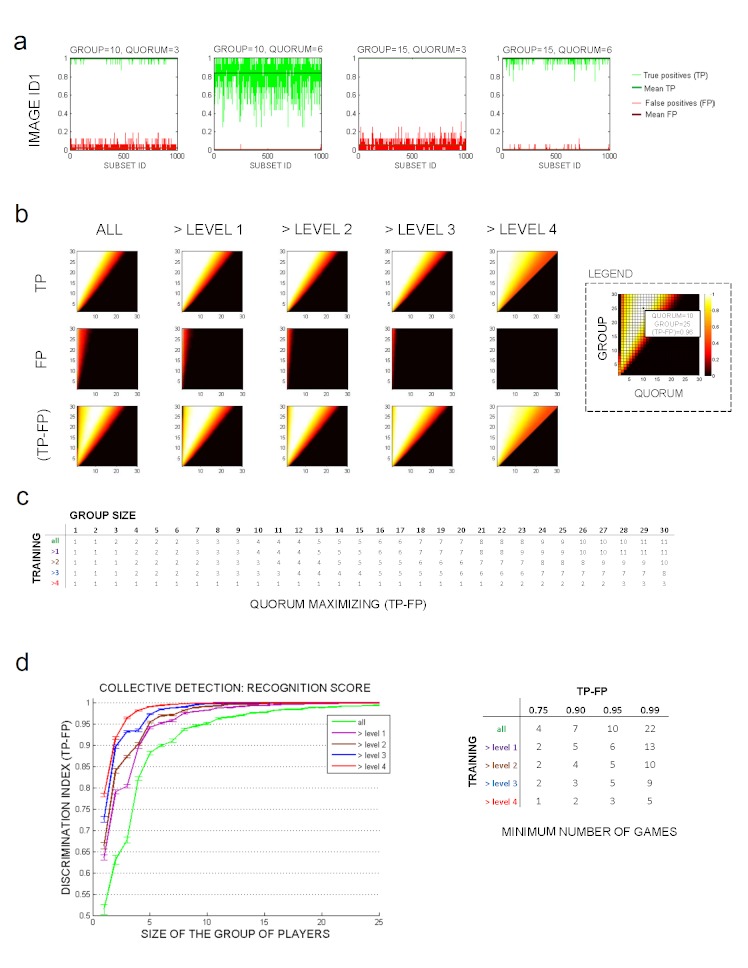
Collective parasite detection. (a) Accuracy results in 1000 random groups run over image ID1 with a group of 10 and 15 games and a quorum of 3 and 6 votes. (b) Mean results of true positives (TP), false positives (FP), and TP – FP for 1000 experiments of all the group sizes and quorum values with different experience. (c) Quorum values that maximize the TP – FP rate for all group sizes and training levels. (d) Maximum recognition score for each group size and training level. Values represent the mean and standard deviation among the regular test images.

We performed an exhaustive evaluation of the collective gamers performance using the quorum algorithm evaluated 1000 times for all group sizes ranging from 1 to 30 games over each of the test images under the different training conditions (completing 1 level can be considered as a 1-minute training) (Figure 5a). Results show a monotone smooth behavior for the true positive (TP) and false positive (FP) rates depending on the group size and quorum value: the bigger the group size or the smaller the quorum required, the more true parasites were tagged and the higher the TP rate, but also more phantom parasites were collectively tagged, increasing the FP rate (Figure 5b). Analysis of the discrimination index (DI) function (DI = TP – FP) revealed that there was an optimal quorum number that maximized the DI for each group size (Figure 5c). For instance, the optimal quorum value was 3 for a group size of 7 games (randomly chosen among all games) achieving a DI = 90%, whereas the optimal quorum for a group of 10 games was 4, providing a mean DI = 95%. When comparing the performance of the collective analysis based on the training time (levels completed), we observed a clear dependence between training and DI (Figure 5d). The number of games needed to be combined in order to achieve a DI = 99% was 22, 13, 10, 9, and 4, respectively, for the subset of games that successfully completed 0, 1, 2, 3, and 4 game levels.

**Figure 6:**
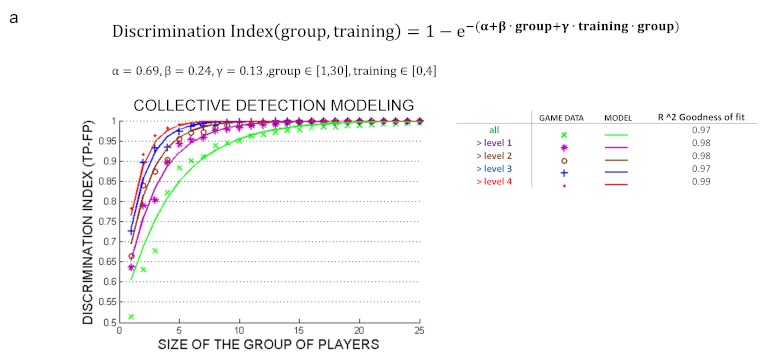
Model of the collective detection of malaria parasites. Curve fitting of the mean accuracy rate for all group sizes and training levels.

The maximum DI for each group size (obtained with its optimal quorum value) at all training levels was fitted to a model equation DI = f(group size, training time) using the scientific data mining software Eureqa [29]. A multivariate optimization process was used to find the following collective detection equation:

*DI(group,training) = 1 - e^–(alpha + beta group + gamma group training)^*

where group size ranged from 1 to 30 games and the coefficient of determination (R^2^) goodness of fit with (alpha = 0.69, beta = 0.24, and gamma = 0.13) is greater than 0.97 for each training value {0,1,2,3,4} levels (or minutes) (Figure 6). This equation highlights the product group⋅training, meaning that the accuracy increase provided by adding 1 game to the group size can almost be compensated (the term beta⋅group varies when increasing the group size) by 1 minute training or vice versa.

We also evaluated the collective performance detection against the automated image recognition methodology presented by Frean [20]. For each image, we calculated the minimum number of gamers needed to perform as well as the automatic system and we found that it was required to combine 7.2, 4.6, 3.9, 3.0, and 2.3 games, respectively, from the subset of players that successfully completed 0, 1, 2, 3, and 4 game levels (see Multimedia Appendix 2).

## Discussion

In this study, a crowdcomputing image analysis system was developed that identifies malaria parasites in digitized images of thick blood smears using an online game played by nonexpert volunteers. Results for the images analyzed showed that the performance of the quorum algorithm that combines the games from different players can be as high as both human expert counting and automated processing methodologies. Indeed, the results showed that, on average, the combination of 22 games or more, regardless of the players’ experience, was enough to obtain almost perfect parasite counting (99%) in the tested images. This performance could also be obtained by combining 13 games from players trained for 1 minute. However, no conclusions could be drawn about detecting extremely low parasitemias (as low as 1 parasite per 30 or more images).

Feedback from several players stressed the need for clearer game instructions. Although our strategy was learning by doing, commenters suggested that an explanation screen and/or an initial training level would lead to better results, at least for those players who did not complete the first level. The collective detection equation allowed us to model the system performance in terms of the number of games and the training of the players. An important question arising from this model is whether any crowdsourced image analysis system that roughly consists of detecting spots in images will have a similar behavior. In the affirmative case, the model could be used in the future to design and evaluate new crowdsourced biomedical image analysis applications.

The overall results endorse the online gaming approach to the task of counting malaria parasites in thick blood films using a crowdsourcing methodology, validated “in the wild” by thousands of anonymous online players. This conclusion extends the findings of Mavandadi et al [25], but from a different perspective. The methodology of the present research involves finding parasites in images from thick blood samples whereas in the study by Mavandadi et al the main task was to make binary decisions (infected versus uninfected) of single-cell images extracted from thin blood samples [25]. Both thin and thick blood films are used in malaria microscopy [26]. The thick film, consisting of many layers of red and white blood cells, is used to search for malaria parasites and to count them as an indicator of the severity of the disease. The thin film, a single layer of red and white blood cells, is mainly used to confirm the malaria parasite species and sometimes to enumerate parasites and evaluate other prognostic features. In addition to the type of data analyzed (thin versus thick blood film) and the task required of the participants (binary decisions in single-cell images versus parasite detection and location), the nature and number of participants varies, from 31 controlled volunteers [25] versus > 6000 anonymous online contributors in the current research. The studies can be considered complementary and directed toward the same goal; the different methodologies and experiments lead to the global conclusion that nonexperts are able to rapidly learn and identify the typical features of malaria parasites in digitized thin and thick blood films, and that the combination of the analyses of several users can provide similar accuracy rates for parasite quantification as expert microscopists.

Future developments of the current research should include the exploration of new algorithms that combine the games of several players in a more complex way than the quorum algorithm. For instance, if players’ identities are logged and tracked, it would be possible to adapt algorithms to differentially weight the analysis of players depending on their profile, experience, past performance, or gaming strategies [30-33]. Automated processing methodologies report accuracy rates that are high, but still not as good as human visual inspection; therefore, we expect that combined man-machine diagnosis systems will be the most effective strategy. Note that, for instance, in the present study the players’ detection performance was similar for all the images, whereas the automated detection algorithm [20] had heterogeneous performances for different images. Therefore, in hybrid systems, humans could be used first to train the recognition algorithm and later to analyze the more complex cases (supplementing the automatic processing methodologies), whereas the easy cases would be automatically processed.

In summary, this proof-of-concept research has shown that malaria image analysis for parasite quantification, obtained by combining the detection of several online nonexperts with minimal training, can be as good as the results provided by an expert microscopist. Although the game score is generated by comparing the user tags with previously analyzed images, in future, the observation protocol from expert microscopists could theoretically be translated into a game and images that have not yet been assessed by professionals could be introduced into that game. This raises the possibility of establishing a global specialized task force of remote gamers-workers able to perform online malaria parasite detection and quantitation. The validity of this approach for malaria diagnosis is still unclear and will depend on the method’s speed, efficiency, robustness, cost, and above all, accuracy. Constraints related to production of the high-quality images required for malaria species identification will have to be addressed. Specifically, the performance should be compared to current diagnostic tools and trends, such as rapid immunochromatographic diagnostic tests that offer a cost-efficient solution. However, rapid tests have limitations, such as restricted malaria species recognition and an inability to quantitate parasite load and monitor parasitological response to treatment. In general, we suggest that the methodology presented in this research could be applied to other biomedical image analysis tasks with potential impact on global health challenges, such as enumeration of acid-fast bacilli in sputum smears for tuberculosis diagnosis. An inherent benefit from this distributed telediagnosis system is that it is scalable and resilient. Among other positive externalities of this research, there is a clear educational impact because more than 6000 players have learned how malaria parasites appear in thick blood films. In addition, as we allowed players to introduce their nicknames into the table of high scorers, we could identify approximately 100 players who now can be considered as experts in parasite counting, within the system’s limitations. Citizen science projects of this kind could impact future educational paradigms: they are a clear opportunity for engaging with young people and offer a hands-on experience that could be used in online learning platforms [34,35].

Concerning the evolution of the MalariaSpot platform, next steps might explore the feasibility of developing a new game version that mimics, if possible, all the relevant steps of the microscopist protocol in real-life conditions (eg, decisions about presence or absence of malaria parasites, parasite stages and species, and quantitation), but this is a much more complex and challenging process. Assuming image quality concerns can be addressed, this system could potentially be completed by integrating the online platform for rapid diagnosis with the recently developed cellphone-microscope systems [36] that allow data transfer directly from field workers and health centers, distributing the data worldwide through the Internet.

## Multimedia Appendix 1

Raw data collected during the experiment: Data_MalariaSpot.csv containing 270207 rows - 1 per parasite tag- in the format: [user id, image id, true/phantom parasite, x position, y position, time].

## Multimedia Appendix 2

Comparison between crowdsourced parasite counting and automatic image analysis counting methodology.

## Abbreviations

DI: discrimination index
FP: false positive
TP: true positive
