# Supplementary material for: Crowdsourcing Malaria Parasite Quantification: An Online Game for Analyzing Images of Infected Thick Blood Smears
Source: J Med Internet Res. 2012 Nov 29;14(6):e167. doi: 10.2196/jmir.2338 (PMC3510720; doi:10.2196/jmir.2338)
Supplement: Supplementary file 2 [file jmir_v14i6e167_app2.pdf]

Multimedia Appendix 2

In *"Reliable enumeration of malaria parasites in thick blood films using digital image analysis"* [20], a digital image analysis methodology for performing parasite density quantitation is reported. The image analysis pipeline combines thresholding techniques and mathematical morphology operators using ImageJ open-source libraries and was evaluated against manual parasite counts in 497 images. Results provided high correlation rates between manual and automatic counting for thick blood films with medium to high parasite densities, and slightly less accurate results for low parasites densities (<6 parasites per image).

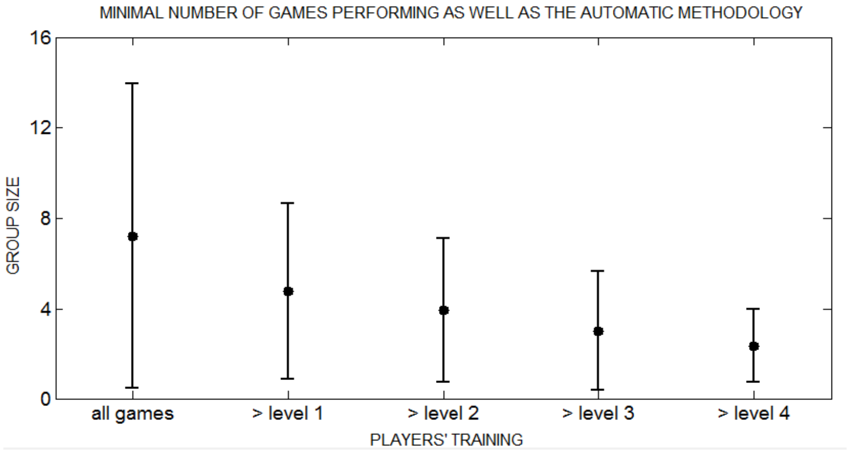

Figure: Group size required to perform as well as an automated parasite counting methodology. The images analyzed here belong to the image group ID BF8A with low-medium parasite density (13.76±3.7 parasites per image), which for the automated counting methodology proposed in [20] provides a mean accuracy of 79±19%. The figure shows the mean and standard deviation number of games needed to be combined using the crowdsourcing analysis and the Quorum algorithm in order to perform as well as the automatic methodology.
